# Technical Quality of Root Fillings Performed by Undergraduate Students: A Radiographic Study

**DOI:** 10.1155/2014/751274

**Published:** 2014-01-28

**Authors:** Tatjana Vukadinov, Larisa Blažić, Ivana Kantardžić, Tijana Lainović

**Affiliations:** ^1^School of Dentistry, Faculty of Medicine, University of Novi Sad, Hajduk Veljkova 3, 21000 Novi Sad, Serbia; ^2^Department of Restorative Dentistry and Endodontics, Clinic of Dentistry of Vojvodina, Hajduk Veljkova 12, 21000 Novi Sad, Serbia

## Abstract

*Aim*. The aim of this study was to evaluate the radiographic technical quality of endodontic treatment performed by undergraduate students at the School of Dentistry, Faculty of Medicine, University of Novi Sad, Serbia. *Materials and Methods*. Electronic records of 220 patients treated by final-year undergraduate students during the school year 2011/2012 were examined, and the final sample consisted of 212 patients, 322 teeth, and 565 root canals. The criteria for overall radiographic adequacy of root canal fillings were defined as the presence of adequate length and density and absence of iatrogenic errors (ledge, fractured instrument, untreated canal, and apical transportation). Chi-square test was used to determine statistical significance between different parameters. *Results*. Adequate root canal fillings were found in 74.22% of the teeth. The percentage of root fillings with adequate length and density was 89.73% and 92.6%, respectively. Fractured instruments and ledges were present in 16 root canals (2.8%), while the presence of missed canal and apical transportation was observed in 2 cases, each (0.3%). *Conclusions*. Overall, the technical quality of root canal fillings performed by undergraduate students was satisfactory.

## 1. Introduction

Endodontic therapy represents an important part of oral health care [1]. Although it is a highly predictable and successful procedure [2], several studies have reported a low percentage of technically adequate root fillings (10.9–55%) [1, 3–11].

In everyday clinical practice, the majority of root canal treatments are performed by general practitioners [12]. Therefore, it is of utmost importance that students achieve a certain level of competence over the course of their education through preclinical and clinical courses. Also, the learning process must not end at graduation. On the contrary, it should be continued throughout the entire work span of a dentist [12]. A study on the levels of confidence of final-year dental students at Cardiff University showed that students are significantly less confident in procedures such as root canal treatment, especially on molars, in comparison with simpler procedures [13]. This fact should not come as a surprise since root canal treatment is often challenging even for general practitioners, and sometimes they unwillingly engage in root canal treatment of posterior teeth [14]. A survey on Danish general practitioners showed that the self-assessment of one's skills is often far from realistic [15]. The majority of the practitioners graded their knowledge and endodontic skills as excellent or satisfactory. However, epidemiological studies showed that the technical quality of root canal treatment performed by general practitioners is at a low level (20.8–40%) [1, 4, 8].

Root canal treatment success can be evaluated by radiographic or clinical findings alone, or both [16]. Radiographic evaluation represents a very frequent method of assessment [17]. Several authors [4, 8, 18, 19] reported a lower incidence of apical periodontitis in teeth with adequate root fillings, so this important variable should be taken into consideration when evaluating root canal treatment success.

Radiographic technical quality of root canal treatment is determined by a number of factors. Some of the prominent ones are instrumentation and obturation level, as well as obturation density [17, 20]. Instrumentation and obturation level positioned 0–3 mm from the radiographic root apex is associated with less untoward events in endodontically treated teeth [8, 19, 21, 22]. Obturation density is considered adequate if the root filling is homogenous with no visible voids within or between the filling and the root walls [20]. Also, it is stated that extrusion of endodontic materials and dentine particles into the periapex causes failures of endodontically treated teeth [21]. Likewise, iatrogenic mistakes, such as fractured instruments and apical perforations, are found to be one of the reasons for nonsurgical root canal treatment failure [23]. Further, canal curvature and tooth position influence the final outcome of root canal treatment, since they can hinder proper shaping of the canals [24]. Hence, all of these variables should be taken into consideration when radiographically evaluating the technical adequacy of root canal fillings.

Based on radiographic findings only, the reported success rates of root canal treatment performed by undergraduate students are 10.9–79.47% [3, 5–7, 10, 11, 25]. In most of these studies, technical quality of treatment was estimated as unsatisfactory, and a need for changes in the preclinical program was stated.

No studies addressing this issue have been conducted in Serbia. Hence, the aim of this study was to evaluate the radiographic technical quality of root canal filling performed by undergraduate students at the School of Dentistry in Novi Sad, Serbia.

## 2. Materials and Methods

After receiving approval from the Ethical Board of the School of Dentistry, Faculty of Medicine, University of Novi Sad, Serbia, records of 220 patients treated by final-year undergraduate students during the school year 2011/2012 at the School of Dentistry in Novi Sad were examined, and information about root canal fillings was acquired. Records of all patients younger than 18 years of age were excluded. Records that did not include preoperative and postoperative periapical radiographs of good quality, with the entire length of root and 2 mm of periapical region clearly visible, were also excluded. The final sample consisted of 212 patients, 322 teeth, and 565 root canals.

All patients were treated by the following protocol: after acquiring information about the patient's medical and dental history, local anesthesia was administrated if needed. Afterwards, access preparation was made and the working length was determined using Propex II electronic apex locator (Dentsply Maillefer, Ballaigues, Switzerland). In unclear cases, an additional radiograph with K-file instrument was made to help determine the working length. The shaping technique used was step-back hand instrumentation with K-files of 0.02 taper (VDW GmbH, Munich, Germany) or Ni-Ti flexible files of 0.02 taper (IMD, Shanghai, China) in curved canals. All canals were irrigated with sodium hypochlorite (0.5%). EDTA (Glyde, Dentsply Maillefer, Ballaigues, Switzerland) was used in calcified and narrow canals. All teeth were obturated with gutta-percha points of 0.02 taper (VDW GmbH, Munich, Germany) and AH Plus (Dentsply DeTrey GmbH, Konstanz, Germany) sealer, using cold lateral condensation technique.

Digital radiographs (preoperative and postoperative) were obtained using a Heliodent Vario D3350 (Sirona Dental Systems GmbH, Bensheim, Germany) and automatically included in the patients' electronic records. Radiographs were examined in Kodak Dental Imaging Software version 6.12.10.0-B for Windows (Carestream Health, Inc. 2009). This software provides the option for measuring root lengths and also the distance between the end of the filling and the root apex.

All radiographs were examined independently by two researchers. Afterwards, the results were compared and the researchers came to a consensus. In case of disagreement (27 root canals), a third investigator was asked to evaluate the radiographs and a final agreement was reached. Strength of agreement was measured using Kappa value (<0 less than chance agreement, 0.01–0.20 slight agreement, 0.21–0.40 fair agreement, 0.41–0.60 moderate agreement, 0.61–0.80 substantial agreement, and 0.81–0.99 almost perfect agreement) [26]. The calculated Kappa value was 0.81.

The technical quality of root canal fillings was evaluated by the length and density criteria presented in Table 1, which are similar to those used in studies by Er et al. [7], Barrieshi-Nusair et al. [10], and Unal et al. [25].

The relation of root canal length and density adequacy to canal curvature and tooth position (anterior/posterior) was assessed. Iatrogenic mistakes, such as ledges, apical transportations, missed canals, and fractured instruments, were also taken into consideration.

The curvature of the canals was evaluated in Kodak software by drawing a straight line through the axial aspect of the root canal. If the line intersected the root apex, the canal was considered straight. Otherwise, it was considered curved [3, 10].

The criteria for overall adequacy of root fillings in this study were defined as the presence of adequate length and density and absence of errors (ledge, fractured instrument, untreated canal, and apical transportation). A tooth was considered adequately filled if all its canals were rated as acceptable. The criteria were uniform for all canals, regardless of canal curvature and tooth position (anterior/posterior).

The statistical analysis was made in Statistica v10 (Statsoft Inc., USA). Chi-square test of independence was used to determine statistical significance between different parameters. The significance level was *P* < 0.05.

## 3. Results

In total, this study included 322 root-filled teeth. The total number of root-filled canals was 565, with the predominance of maxillary teeth (62.29%). More than one-third of the samples, 122 (37.89%), were incisors and canines, followed by 103 (31.99%) premolars and 97 (30.12%) molars. The most commonly root-filled teeth were maxillary incisors (17.39%), followed by mandibular premolars (16.15%).

The length and density of the root canal fillings according to the root canal curvature are presented in Table 2. Chi-square tests of independence between the canal curvature and length of the filling showed that curvature is related to length adequacy. Compared with curved canals, root fillings of adequate length were observed in a significantly greater proportion in straight canals (*P* < 0.001). However, no significant difference was observed for the adequacy of density between straight and curved canals (*P* > 0.05).

Tests of independence between the root canal location (maxilla/mandible) and adequacy of the canal filling length showed that tooth location is related to length adequacy (Table 3). Compared to mandibular teeth, the percentage of root fillings with adequate length was significantly greater in maxillary teeth (*P* < 0.05).

However, no dependence was established between the tooth position (anterior/posterior) and canal length adequacy (*P* > 0.05). No statistically significant differences were observed between the densities of maxillary and mandibular canals or between the anterior and posterior canals (*P* > 0.05).

Table 4 presents the length and density of the root fillings according to tooth group. The percentage of root canal fillings with adequate length was 89.73%. The highest percentage of root fillings with adequate length was observed in maxillary canines and premolars (96.67%). The overall percentage of root canal fillings with adequate density was 92.6%, with the best results achieved in mandibular incisors (100%).

Considering the occurrence of iatrogenic errors, fractured instruments and ledges were present in 16 root canals (2.8%), while the presence of missed canal and apical transportation was observed in 2 cases, each (0.3%). However, at the present level of data acquisition, the number of detected errors is still insufficient to allow valid hypotheses tests.

Adequacy of root fillings by tooth group is presented in Table 5. There was no statistically significant difference between the overall adequacy of root canal fillings in maxillary and mandibular teeth. On the other hand, there were significant differences between tooth groups within particular jaws. Compared to maxillary molars, adequate root fillings were found in a significantly greater proportion in maxillary incisors, canines, and premolars (*P* < 0.05). In comparison with mandibular molars, a significantly higher percentage of adequate root fillings was found in mandibular premolars (*P* < 0.05) and mandibular incisors (*P* < 0.05).

Considering the overall adequacy, a total of 450 (80.0%) of the canals, or 74.22% of the 322 teeth, qualified as acceptable.

## 4. Discussion

Radiographic evaluation of the technical quality of root canal fillings performed by undergraduate dental students at the School of Dentistry in Novi Sad was presented in this study. A total of 212 patient charts, 322 teeth, and 565 root canals were evaluated. Routine procedural periapical radiographs were used for this study. Since radiographs are two-dimensional, root fillings or anatomic structures are often superimposed to each other, therefore making it impossible to make a valid assessment of endodontic treatment quality. In epidemiological studies researchers used different criteria for radiographic evaluation. Some used length only [27, 28], while others took length and density [6, 11] or, in addition to the two, even taper into consideration [3, 7, 18]. In this study, the taper of root canal filling was not taken into consideration, since it was concluded that it is a highly subjective criterion [25].

Burke et al. [28] stated in their 5-year follow-up study that the length of root canal filling is the most important factor for survival of endodontically treated teeth. The authors found that pretreatment periapical pathology played no significant role in teeth survival. On the other hand, Chugal et al. [20] stated that the level of root canal preparation is important for treatment success but that preoperative diagnosis is the most important factor. Thus, they concluded that the root canal length must only be considered with preoperative diagnosis. The percentage of root fillings with adequate length in the present study was 89.7%, which is higher than in other studies [3, 5–7, 10, 11]. There are several reasons for the discrepancy in the results. Firstly, the length measured on periapical radiographs is often inaccurate, so there is a certain threshold of what is considered to be a correct filling. Some authors stated a 0–2 mm distance from the end of the filling to the root apex as adequate, while others set this limit at 0–3 mm. In the Quality Guidelines for Endodontic Treatment [29], it is stated that if the tip of the instrument during radiographic root canal measurement is ≤3 mm away from the radiographic root apex, there is no need for further working length adjustments. In this study, the 0–3 mm threshold was considered adequate, as had been done in several previous studies [8, 19, 21, 22]. Secondly, students at the School of Dentistry in Novi Sad use an electronic apex locator on regular bases. If working length is inconclusive, additional radiographs with a K-file instrument are made.

The present work showed a statistically significant correlation between filling length and the curvature of canals, which supports findings of several other studies [3, 7, 10]. There are significantly more root canal fillings of inadequate length in curved than in straight canals. Additionally, the endodontic filling length was adequate statistically more often in maxillary than in mandibular teeth, similar to the study by Khabbaz et al. [11].

Adequate density of root canal filling is an important factor for long-term success of endodontic treatment [30, 31]. In this study, 92.6% of canals were of adequate density. This result is higher compared to other studies which reported 27.6%–72.6% of canals with adequate density [3, 5–7, 10, 11]. In contrast to the study by Moussa-Badran et al. [6], in this study there was no significant correlation between tooth type and density of the filling, although slightly different criteria were used, which makes comparison difficult. Undergraduate students at the Dental School in Novi Sad use cold lateral condensation technique with gutta-percha points and AH Plus sealer. Lateral condensation technique is in use in numerous dental teaching centres across Europe and The United States [32, 33], although single cone techniques are less time consuming [34].

Procedural errors are an important factor for long-term survival of endodontically treated teeth. They lead to inadequate instrumentation and/or obturation of the canals [35]. In this work there were 3.4% of procedural errors in total. Ledges occurred in 2.8% of the cases, while fractured instruments were found in 0.3% of the cases. This differs significantly from results in a study by Khabbaz et al. [11], where the investigators found ledges in 54.8% of canals and separated instruments in 0.9% of the canals. Also, Balto et al. [5] reported 13.6% of ledges and 0.5% fractured instruments, respectively. Contrary to the present study, Khabbaz et al. [11] did not find any missed canals, but they reported root and apical foramen perforation in 11.8% and 32.6% of the canals, respectively. In a study by Rafeek et al. [3] 1.5% of fractured instruments were reported, which is again considerably higher compared to this study. Such a discrepancy in the number of ledges found in the present study and similar studies could be due to interexaminer variation and to the fact that it is difficult to set a unique definition of a ledge. Nevertheless, one of the reasons why students at the School of Dentistry in Novi Sad achieved such a low level of iatrogenic errors was the fact that they used Ni-Ti flexible instruments and EDTA in curved canals, which prevented them from creating ledges. It has been shown that the use of stainless steel files in such canals leads to the creation of ledges [36].

The present study shows that undergraduate students at the School of Dentistry in Novi Sad have a satisfactory success in root canal filling of anterior and posterior teeth. Nevertheless, their results could be improved. The overall radiographic adequacy of root canal fillings performed by final-year undergraduate dental students was 74.22%, which is similar to 79.47% reported by Unal et al. [25] and higher than 10.9–55% reported by several other authors [3, 5–7, 10, 11]. The criteria used in these studies were slightly different; therefore they cannot be fully compared. In this study, the researchers used a wider limit for adequacy of root canal length than the researchers stated above. Nevertheless, the results of the present work remain at a high level even compared to studies that used the same length criteria, such as 65% in a study by Sidaravicius et al. [21] and 26.52% reported by Kirkevang et al. [19]. Satisfactory results in technical quality of root canal treatment achieved by final-year students at the School of Dentistry in Novi Sad might be a result of a combination of factors stated earlier in the text, such as the use of electronic apex locators, flexible instruments in curved canals, and immediate radiographic evaluation. Also, the influence of preclinical and clinical endodontics course and teaching methods used therein can be assumed to be very strong.

Mastering theoretical knowledge of main principles in endodontics, as well as preclinical practice, is of utmost importance before undertaking clinical practice [12]. At the School of Dentistry in Novi Sad, students participate in lectures and practical training in endodontics during the 8th, 9th, and 10th semester of undergraduate studies. In the 8th semester, the course *Endodontics I* includes one class of lectures and two practical classes per week. During this period, students are required to learn the basic principles of endodontic therapy and practice on cylindrical endotrainers (frasaco GmbH, Tettnang, Germany) and extracted teeth. Endodontic treatment of molar teeth is often difficult due to their complex root canal anatomy, so training on this tooth group should be amplified. At the end of the semester, students are required to pass a written exam in order to progress to the course *Endodontics II*. This course includes 15 classes of lectures and 180 classes of clinical practice during 9th and 10th semester. Clinical practice is held by specialists in Tooth Disease and endodontics at a 1-to-8 teacher-to-student ratio. In order to improve preclinical and clinical teaching in endodontics, it would be useful to incorporate self-assessment of the quality of endodontic treatment in the curriculum, as students should be able to judge the quality of their own work and maintain or improve that quality level after they graduate.

## 5. Conclusion

Within the limitations of the presented study, it can be concluded that 74.22% of root canal fillings performed by final-year undergraduate students at the School of Dentistry in Novi Sad were radiographically adequate, which is satisfactory given the students' lack of experience.

## Figures and Tables

**Table 1:** Criteria for evaluation of root canal fillings.

Parameters	Criteria	Description
Length	Acceptable	Root filling ends 0–3 mm from the root apex
	Overextended	Root filling is extruded in the periapex
	Short filling	Root filling ends > 3 mm from the root apex
Density	Adequate	Root filling is homogenous with no visible voids within or between the filling and the root walls
	Inadequate	Root filling is not homogenous with visible voids within or between the filling and the root walls

**Table 2:** Length and density of root canal fillings in straight and curved canals.

	Root canal	Length			Density	
		Adequate	Overextended	Short	Adequate	Inadequate
Straight	411 (72.74%)	387 (94.16%)*	7 (1.70%)	17 (4.14%)	379 (92.21%)^†^	32 (7.99%)
Curved	154 (27.26%)	120 (77.92%)	3 (1.95%)	31 (20.13%)	144 (93.51%)	10 (6.49%)

Total	565 (100%)	507 (89.73%)	10 (1.77%)	48 (8.50%)	523 (92.57%)	42 (7.43%)

*Statistically significant difference (*P* < 0.01) between adequate lengths of root canal fillings in straight and curved root canals.

^†^No statistically significant difference (*P* > 0.05) between adequate densities of root canal fillings in straight and curved root canals.

**Table 3:** Length and density of root fillings by canal location (maxilla/mandible).

Canal location	Total	Length			Density	
		Adequate	Overextended	Short	Adequate	Inadequate
Maxillary canals	316 (55.93%)	293 (92.72%)*	4 (1.26%)	19 (6.02%)	292 (92.40%)^†^	24 (7.60%)
Mandibular canals	249 (44.07%)	214 (85.94%)	6 (2.41%)	29 (11.65%)	231 (92.77%)	18 (7.23%)

Total	565 (100%)	507 (89.7%)	10 (1.8%)	48 (8.5%)	523 (92.6%)	42 (7.4%)

*Statistically significant difference (*P* < 0.05) between adequate lengths of root canal fillings in maxillary and mandibular teeth.

^†^No statistically significant difference (*P* > 0.05) between adequate density of root canal fillings in maxillary and mandibular teeth.

**Table 4:** Length and density of root canal fillings by tooth group.

Tooth group	Total	Length			Density	
		Adequate	Overextended	Short	Adequate	Inadequate
Maxilla						
Incisors	55 (9.73%)	52 (94.55%)	1 (1.82%)	2 (3.64%)	53 (96.36%)	2 (3.64%)
Canines	30 (5.31%)	29 (96.67%)	0 (0.0%)	1 (3.33%)	28 (93.33%)	2 (6.67%)
Premolars	90 (15.93%)	87 (96.67%)	0 (0.0%)	3 (3.33%)	87 (96.67%)	3 (3.33%)
Molars	141 (24.96%)	125 (88.65%)	3 (2.13%)	13 (9.22%)	124 (87.94%)	17 (12.06%)
Mandible						
Incisors	21 (3.72%)	20 (95.24%)	1 (4.76%)	0 (0.00%)	21 (100.0%)	0 (0.0%)
Canines	17 (3.00%)	16 (94.12%)	0 (0.00%)	1 (5.88%)	14 (82.35%)	3 (17.65%)
Premolars	58 (10.27%)	54 (93.10%)	1 (1.72%)	3 (5.17%)	53 (91.38%)	5 (8.62%)
Molars	153 (27.08%)	124 (81.05%)	4 (2.61%)	25 (16.34%)	143 (93.46%)	10 (6.54%)

Total	565 (100%)	507 (89.73%)	10 (1.77%)	48 (8.50%)	523 (92.6%)	42 (7.4%)

**Table 5:** Adequacy of tooth fillings by tooth group.

Tooth group	Total	Acceptable	Unacceptable
Maxilla			
Incisors	56 (17.39%)	47 (83.93%)*	9 (16.07%)
Canines	31 (9.63%)	26 (83.87%)^†^	5 (16.13%)
Premolars	51 (15.84%)	39 (76.47%)^‡^	12 (23.53%)
Molars	46 (14.29%)	25 (54.35%)	21 (45.65%)
Mandible			
Incisors	20 (6.21%)	18 (90.00%)^§^	2 (10.00%)
Canines	15 (4.65%)	12 (80.00%)	3 (20.00%)
Premolars	52 (16.15%)	42 (80.77%)^II^	10 (19.23%)
Molars	51 (15.84%)	30 (58.82%)	21 (41.18%)

Total	322 (100.0%)	239 (74.22%)	83 (25.78%)

*Statistically significant difference (*P* < 0.05) between acceptable tooth fillings in maxillary incisors and molars.

^†^Statistically significant difference (*P* < 0.05) between acceptable tooth fillings in maxillary canines and molars.

^‡^Statistically significant difference (*P* < 0.05) between acceptable tooth fillings in maxillary premolars and molars.

^§^Statistically significant difference (*P* < 0.05) between acceptable tooth fillings in mandibular incisors and molars.

^II^Statistically significant difference (*P* < 0.01) between acceptable tooth fillings in mandibular premolars and molars.
